# Toxic effects of antimony in plants: Reasons and remediation possibilities—A review and future prospects

**DOI:** 10.3389/fpls.2022.1011945

**Published:** 2022-10-26

**Authors:** Haiying Tang, Guiyuan Meng, Junqing Xiang, Athar Mahmood, Guohong Xiang, SanaUllah, Ying Liu, Guoqin Huang

**Affiliations:** ^1^ College of Agriculture and Biotechnology, Hunan University of Humanities, Science and Technology, Loudi, China; ^2^ Loudi Liancheng Hi-Tech Agricultural Development Co. LTD, Loudi, China; ^3^ Department of Agronomy, University of Agriculture Faisalabad, Faisalabad, Pakistan; ^4^ Agronomic Research Station Karor, Layyah, Pakistan; ^5^ Key Laboratory of Crop Physiology, Ecology and Genetics Breeding (Jiangxi Agricultural University), Ministry of Education, Nanchang, China; ^6^ Research Center on Ecological Sciences, Jiangxi Agricultural University, Nanchang, China

**Keywords:** antimony, growth, health risks, photosynthesis, remediation

## Abstract

Antimony (Sb) is a dangerous heavy metal (HM) that poses a serious threat to the health of plants, animals, and humans. Leaching from mining wastes and weathering of sulfide ores are the major ways of introducing Sb into our soils and aquatic environments. Crops grown on Sb-contaminated soils are a major reason of Sb entry into humans by eating Sb-contaminated foods. Sb toxicity in plants reduces seed germination and root and shoot growth, and causes substantial reduction in plant growth and final productions. Moreover, Sb also induces chlorosis, causes damage to the photosynthetic apparatus, reduces membrane stability and nutrient uptake, and increases oxidative stress by increasing reactive oxygen species, thereby reducing plant growth and development. The threats induced by Sb toxicity and Sb concentration in soils are increasing day by day, which would be a major risk to crop production and human health. Additionally, the lack of appropriate measures regarding the remediation of Sb-contaminated soils will further intensify the current situation. Therefore, future research must be aimed at devising appropriate measures to mitigate the hazardous impacts of Sb toxicity on plants, humans, and the environment and to prevent the entry of Sb into our ecosystem. We have also described the various strategies to remediate Sb-contaminated soils to prevent its entry into the human food chain. Additionally, we also identified the various research gaps that must be addressed in future research programs. We believe that this review will help readers to develop the appropriate measures to minimize the toxic effects of Sb and its entry into our ecosystem. This will ensure the proper food production on Sb-contaminated soils.

## Introduction

Rapid industrial development and intensive agricultural practices have substantially increased the concentration of heavy metals (HMs) in our environment (Hasssan et al., 2020; Rasheed et al., 2021a; Rasheed et al., 2021b). HMs are naturally occurring elements with atomic weight and density at least five times greater than water (Tchounwou et al., 2012). HM pollution has become a widespread problem globally, and it is negatively affecting human health, crop productivity, and soil health (Hasssan et al., 2020; Rasheed et al., 2020; Rasheed et al., 2020a; Rasheed et al., 2020b). Among different industries, mining and smelting of non-ferrous metals are considered the main sources of HM pollutants in our environment (Xing et al., 2020). Among different HMs, antimony (Sb) has emerged as a serious toxic metal, and its concentration is also increasing in our soil owing to anthropogenic activities (Ma et al., 2019). It is used in various industrial products, and it is a trace metal and considered to be toxic for humans and plants (Chai et al., 2016; Ma et al., 2019). The excessive intake of Sb in humans through eating Sb-contaminated foods can cause cancer, liver, and cardiovascular diseases (Herath et al., 2017; Jamali et al., 2017). Once Sb enters into the human body, it also reacts with sulfhydryls and disturbs the enzymatic reactions thus leading to cellular hypoxia (Yang et al., 2015). Due to its rising concentration and toxic effects, Sb has been listed as a top pollutant by the European Union and USA environmental protection agency (Feng et al., 2020).

**Figure 1:**
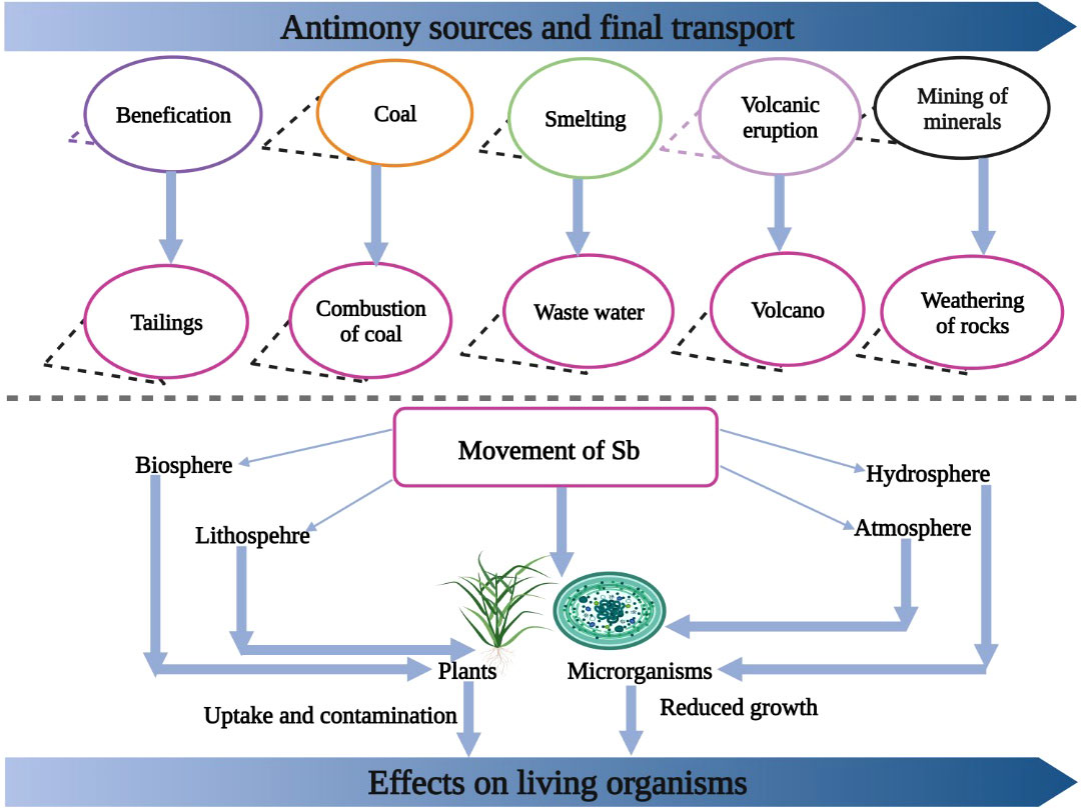
The various sources of Sb entry into environment. Sb enters into environment from burning of coal, smelting, volcanic eruptions and mining of minerals. After entering into environment it cause toxic effects to plants and humans.

Volcanic activities and rock weathering are natural sources of Sb in our environment (**Figure 1**); however, these processes release little amount of Sb into the environment (Baroni et al., 2000). Nonetheless, human activities including smelting, mining, and fossil burning release a large quantity of Sb, which is causing a serious threat of Sb in many parts of the world (Tschan et al., 2009). China has the largest reserves of Sb accounting for >90% of the world’s Sb production, followed by Australia, Russia, South Africa, Tajikistan, Canada, and the USA (Corrales et al., 2014; Miao et al., 2014). The concentration of Sb in Hunan province of China and in Italy has increased to 5045 and 4400 mg/kg, respectively (He, 2007; Corrales et al., 2014). Sb is non-essential; however, it is readily absorbed by plants, which can cause plant death (Feng et al., 2020).

It is considered that Sb concentration in soils greater than 150 mg/kg causes damage to plants (Feng et al., 2020). It affects all plant processes ranging from germination, growth, development, photosynthesis, and induced reactive oxygen species (ROS) production; all these changes induce a serious reduction in plant growth (Remans et al., 2012; Zeng et al., 2015). Sb present in soil solution is readily absorbed by plant roots, which, in turn, reduces growth, photosynthesis, and synthesis of proteins and metabolites (Feng et al., 2013b). In addition, Sb also reduces nutrient uptake, consequently reducing biomass and growth of plants (Cai et al., 2016). The high concentration of Sb in soils and sediments is toxic to ecosystems and the human health (Shahid et al., 2018). The acceptable levels of Sb in water and soil are 0.020 mg L-^1^ and 36 mg kg^-1^, respectively (Guo et al., 2009), and an increase in the Sb concentration above these levels causes serious damage to plants and humans. Plants have developed a promising antioxidant system to cope with the damage of Sb toxicity (Bolan et al., 2022). Plants also accumulate various osmolytes and secondary metabolites to counter the effects of HMs (Bolan et al., 2022).

Immobilization, mobilization, bioremediation, and phytoremediation practices are taken for the remediation of soils contaminated with Sb (Hua et al., 2021). A wide range of materials, including carbon-based biocomposites, biochar, nanoparticles, mineral sorbents, and imprinted polymers, have been identified to mitigate the damaging effects of Sb (Jia et al., 2020). The use of various amendments can appreciably reduce the toxicity and bioavailability of Sb contamination (Long et al., 2020). Recently, it has been recognized that Sb remediation can also be done by various biological and physicochemical approaches (Nishad and Bhaskarapillai, 2021). Therefore, in the present review, we systematically discussed the various sources of Sb in the environment and its toxic effects on plants and humans. Moreover, this review also presents recent developments to understand the role of various amendments to mitigate Sb toxicity. Additionally, we also identified the various research gaps that must be fulfilled in future research studies to mitigate Sb toxicity.

## Sources of antimony in environment

Sb is present in soil (**Table 1**), water, and air, and it is present in lower concentrations (0.2–0.3 μg g^-1^) compared with other HMs (Hiller et al., 2012; Fort et al., 2016). Generally, the concentration of Sb in rocks is around 0.2 μg g^-1^; however, in shale rocks, the concentration of Sb can reach up to 300 μg g^-1^ (Zhuang et al., 2018). Sb is present in organic as well as inorganic forms in the environment (Vikent’Eva and Vikentev, 2016; Zhuang et al., 2018), and Sb concentrations vary in diverse environments owing to leaching from rocks, ores, and biogeochemical conditions (Okkenhaug et al., 2016). Moreover, Sb is also present in deposits of clay minerals, and it also has an association with coal organic matter (Qi et al., 2008). Generally, pentavalent and trivalent forms of Sb are more lethal, and they are present in natural environments (Coughlin et al., 2020). Generally, the concentration of Sb in water is < 1 μg mL^−1^, whereas, in soils, the concentration of Sb is in the range 0.3–8.6 mg kg^-1^ (Pierart et al., 2015). Like other elements, the concentration of Sb in any environment also depends on the parent materials (Tschan et al., 2009). The concentration of Sb in clean and sea water is <1 and 0.2 μg L^-1^, respectively, whereas in contaminated water, Sb is present up to 100 μg L^-1^ (Filella et al., 2002). According to the World Health Organization (WHO), the acceptable value of Sb in potable water is 5.0 μg L^-1^, whereas according to Australian drinking water guidelines, the Sb concentration in potable water should not be >3 μg L^-1^ ((NHMRC and NRMMC, 2011).

**Table 1:** Concentration of Sb in soils of different countries.

Country	Sb Concentration (mg kg^-1^)	Site	Reference
Australia	22000	Wetlands	(Warnken et al., 2017)
Australia	2735	Mining area	(Wilson et al., 2013)
China	547	Mining area	(Couto et al., 2015)
Czech Republic	131	Agriculture soil	(Ettler et al., 2010)
Germany	1.75	Urban soil	(Thestorf and Makki, 2021)
Italy	4400	Mining site	(Cidu et al., 2014)
Japan	2900	Smelting site	(Takaoka et al., 2005)
Korea	67.48	Firing range area	(Ahmad et al., 2014)
Mongolia	55.20	Mining area	(Qi et al., 2008)
New Zealand	80200	Smelting site	(Wilson et al., 2004)
Poland	499	Mining site	(Lewińska and Karczewska, 2019)
Scotland	1200	Mining site	(Gal et al., 2007)
Spain	1,090	Mining site	(Murciego et al., 2007)
Vietnam	95	Mining site	(Cappuyns et al., 2021)

Metal mining and pharmaceutical manufacturing are the primary sources of the toxic form of Sb [(Sb(V)] (Zhuang et al., 2018). Similarly, sewage sludge, emissions from the vehicle, leaching from plastic waste, industrial dumps, and direct infiltration from solid wastes are also important sources of Sb in the environment (Chu et al., 2021; Hu et al., 2021). Moreover, Sb alloys and its compounds are used to prepare conductors, batteries, pesticides, solder alloys, and fireworks, which are important sources of Sb in our environment (Diquattro et al., 2021). About 2–8% Sb is also used to harden the bullets utilized in shooting (Mariussen et al., 2017), and when these bullets enter into the soil, they increase the accumulation of lead (Pb) and Sb in soils. It has been documented that 12 tons of Sb is accumulated per annum at the military shooting range of Norway, which is a major source of Sb in Norway (Mariussen et al., 2017).

Sb is also being used in plastic products (Chu et al., 2021), and it has been recorded that Sb is present in daily-use items like foams, fibers, and rubbers (Turner and Filella, 2017). In 2020, the global Sb production stood at 153,000 tonnes, and 14% of this production was used by the US Geological Survey (2021). A large quantity of Sb is released into the environment everyday due to its substantial use in diverse products (Hu et al., 2021) that is posing a serious threat to human health, the environment, and plants (He et al., 2019b). Polyethylene terephthalate (PET) fabrication is also a major source of Sb release into the environment (Chu et al., 2021). The disposal of PET waste fibers released 1108 tons of Sb into landfills, whereas chemical, incineration, and mechanical processes released 25, 284, and 794 tonnes of Sb, respectively, into the environment (Chu et al., 2021). The historic Sb-mine sites with poorly managed waste are also a significant source of Sb release into the environment (Intrakamhaeng et al., 2020).

Sb precipitation also induces its mobilization into ground and surface waters (**Table 2**), which diminishes the quality of drinking water (Mbadugha et al., 2020). The crops accumulate a huge quantity of Sb, which, in turn, enters into the human food chain resulting in various health problems in humans (Mbadugha et al., 2020). The level of Sb is considered to be decreased with the increase in the distance from the mining as well as processing zones (Macgregor et al., 2015). The contamination of ground water with Sb results from various activities including weathering of parent materials, leaching, wet deposition, mining, pesticide application, and industrial effluents (Etim, 2017). In ground water, Sb exists in trivalent as well as pentavalent forms (Etim, 2017), and these forms undergo oxidation and reduction. The soluble form of Sb is transferred into water, while its less soluble form is absorbed by the clay fragments (Etim, 2017). Moreover, Sb also leaches from the landfills and sewage sludge, and it is then transported into ground and surface waters and causes serious health problems in humans (Campos et al., 2019; Intrakamhaeng et al., 2020). Additionally, Sb leaches into sediments by microbial actions, which is also a major reason for Sb pollution (Intrakamhaeng et al., 2020).

**Table 2:** Concentration Sb of in water bodies of different countries.

Country	Sb Concentration (mg L^-1^)	Site	Reference
China	0.038	Mining water	(Qiao et al., 2018)
France	0.0067-0.156	Mining water	(Thestorf and Makki, 2021)
Ghana	0.75	Mining water	(Serfor-Armah et al., 2006))
Italy	19-4400	Sb abandoned mines	(Cidu et al., 2014)
Iran	0.191	Sb mining area	(Ghassemzadeh et al., 2006)
Korea	0.080	Mining water	(Jo et al., 2018)
Norway	19-349	Shooting range	(Mariussen et al., 2017)
Slovakia	9861	Sb abandoned mines	(Hiller et al., 2012)
Turkey	0.271	Sb deposits area	(Kurt et al., 2021)

## Antimony guideline values

Sb enters our environment through anthropogenic activities as well as weathering of rocks consisting of Sb mining and smelting activities (He et al., 2019a). Sb and various compounds containing Sb have been recognized as emerging pollutants, which is causing serious damage to humans, plants, and the environment (He et al., 2019a). Prolonged exposure to high levels of Sb through drinking water causes a serious health issue (WHO, 2003). Globally, different countries proposed various guideline values for drinking water with pollutants including Sb to limit their hazardous impacts on humans. There is a significant difference in the guideline values of Sb for water, sediments, and soils globally (Nishad and Bhaskarapillai, 2021). These guidelines have been developed considering the different factors including sociocultural, biological, political, scientific, and geographic factors (Bagherifam et al., 2019). The maximum concentration of Sb in drinking water set by the United States Environmental Protection Agency (USEPA) and the WHO are 6 and 20 ppb, respectively (He et al., 2012; Nishad et al., 2017).

## Toxic effects of antimony on plants

Sb is a non-essential and toxic metal that is readily absorbed by plants and causes a serious reduction in growth. Sb stress reduces chlorophyll synthesis, induces ROS production and MDA accumulation, damages the chloroplast structure, and brings ultrastructural changes in the body, thereby resulting in a substantial reduction in plant growth (Zhou et al., 2018).

## Effect of Sb toxicity on plant growth and development

**Figure 2:**
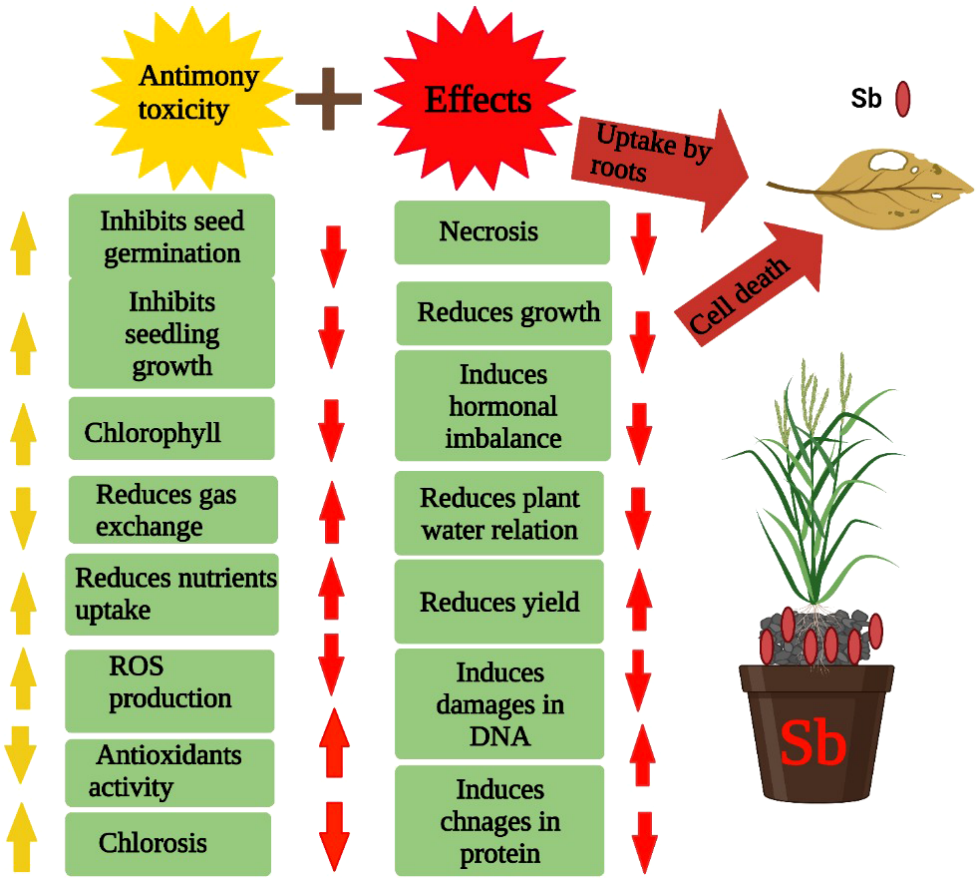
Sb stress reduces the seed germination, seedling growth, chlorophyll contents, nutrient uptake, disturbs hormonal balance, water relations, damage DNA and protein and induce ROS which in turn cause significant reduction in plant growth and development.

Sb present in the soil solution is readily absorbed by plants causing deleterious impact on these plants (Maresca et al., 2020). Sb stress induces growth reduction by decreasing photosynthesis and nutrient uptake (**Figure 2**), and assimilates the production and synthesis of hormones and metabolites (Zhou et al., 2018). Sb toxicity also reduces the growth of plants by decreasing the nutrient uptake and increasing the production of ROS that causes damage to plant membranes, proteins, and lipids (Kamiya and Fujiwara, 2009; Cai et al., 2016). Plant height is an important indicator of growth, and Sb stress significantly reduces the plant height and also induces the development of thinner and smaller leaves. The development of thinner leaves reduces production thus significantly reducing plant growth (Zhou et al., 2018). Beyond a certain level, Sb also inhibits growth and reduces physiological functioning, which is a major reason for Sb-induced growth reduction (Zhou et al., 2018). Plants activate an excellent defense system to cope with the toxic effects of Sb; however, excessive concentration of Sb beyond a certain level weakens the antioxidant defense system and inhibits the growth, plant height, and dry matter production (Zhou et al., 2018). Some plants have good tolerance against Sb stress; however, the effect of Sb can vary amid the plant species and varieties of different plants (Dahmani-Muller et al., 2000; Shtangeeva et al., 2012). Sb toxicity also causes reduction in biomass production, and an increase in Sb toxicity linearly decreased the growth and biomass production (Bech et al., 2012; Shtangeeva et al., 2012). In another study, Pan et al. (2011) documented a serious reduction in root and shoot biomass, root numbers, and root length of plants grown under Sb stress (50 to 1000 mg/kg). Conversely, in maize and sunflower plants, no toxic effects of Sb stress were noted (Tschan et al., 2010). Moreover, a slight reduction in biomass production was observed in *Pteris cretica* L. plants, which indicated a significant difference among plant species for Sb tolerance (Feng et al., 2011b). In conclusion, Sb toxicity negatively affects photosynthesis and assimilates production, nutrient uptake, and synthesis of various growth-promoting hormones, thereby causing a substantial reduction in plant growth.

## Effect of Sb toxicity on nutrient uptake

The exposure of plants to Sb disturbs the uptake and distribution of essential mineral nutrients in plants. For instance, Shtangeeva et al. (2012) recorded that Sb stress (50–150 mg L^-1^) significantly reduced the concentration and uptake of Ca, K, Na, and Cu in wheat plants. Similarly, rice plants treated with Sb (9 mg L^-1^) showed a considerable reduction in Ca, Mg, Fe, Mn, and Zn uptake and accumulation in plant parts (Feng et al., 2013b). A higher level of Sb also decreased the accumulation of soluble proteins owing to a reduction in the uptake of N (Lan et al., 2009). In another study conducted on cabbage plants, it was showed that Sb has a synergistic effect on the accumulation of Cu, Mn, and Zn (Geng et al., 2020). Conversely, in red beet plants, it was noted that Ca and iodine concentration was significantly increased, whereas the concentration of Mn and Zn was considerably decreased, resulting in a significant reduction in plant growth (Geng et al., 2020). The exposure of plants to Sb also affects the trace elements and macronutrients, which further causes toxicity in plants owing to deficiency and excess of these elements (Wilson et al., 2013). In another study, Shtangeeva et al. (2012) noted that Sb significantly reduced Ca, Cu, K, and Na concentration and uptake in wheat plants growing under Sb stress as compared with the control. Moreover, a decrease in Cu, Mg, and Zn concentration in cabbage plants was recorded with Sb stress (Xi-Yuan et al., 2015). Additionally, Ortega et al. (2017) also noted that Sb absorption by plants reduced Cu, Fe, Mg, and Zn uptake and concentration, and this reduction was further intensified with increasing Sb stress.

## Effect of Sb toxicity on photosynthetic pigments and photosynthesis

Photosynthesis is one of the most important processes in plants, and Sb is found to inhibit photosynthesis by decreasing chlorophyll synthesis (Zhang et al., 2010; Pan et al., 2011). In another study, it was found that Sb stress in *Acorus calamus* significantly decreases the chlorophyll and carotenoid levels, results in a significant reduction in photosynthesis, and assimilates production (Zhou et al., 2018). Sb stress decreases the chlorophyll and carotenoid contents by decreasing the biosynthesis of these compounds (Xue et al., 2015). The negative effects of Sb on the synthesis of these compounds are also linked with a reduction in the absorption of Fe and Mg owing to Sb toxicity (Ortega et al., 2017). The decrease in carotenoid contents owing to Sb stress destabilizes thylakoids and causes the failure of ROS elimination resulting in a considerable reduction in photosynthesis (Zhou et al., 2018). Conversely, Sb causes no structural inhibition and damage to PS-II and reduction in photosynthesis in *Ficus tikoua* and *Boehmeria nivea* (Chai et al., 2016; Chai et al., 2017).

Stomatal closing is a key physiological response in plants grown under stress conditions. Sb toxicity induces stomata closing, which reduces CO_2_ intake and results in a substantial reduction in photosynthesis (Romanowska et al., 2006). Sb also causes damage to the meta-xylem vessels and induces stomata closure, thus resulting in a reduction in the photosynthetic rate and efficiency in plants (Baruah et al., 2021). Conversely, Chai et al. (2016) noted that Sb induced no significant effect on the chlorophyll fluorescence parameters of ramie. Sb also decreased the photochemical quantum yield (Fv/Fm) and led to a serious reduction in plant photosynthetic efficiency under Sb stress (Roccotiello et al., 2016; Qiang et al., 2017). The exposure to Sb also inhibits photosystem-II (PS-II), which is also a major reason for the substantial reduction in photosynthesis and subsequently induces a serious reduction in growth (Zhang et al., 2010; Pan et al., 2011).

Vaculík et al. (2015) noted a significant decrease in the chlorophyll contents of sunflower under Sb stress; Sb also alters large units of ribulose-1,5-bisphosphate carboxylase/oxygenase (RuBisCo), which, in turn, induces serious reduction in photosynthesis (Duquesnoy et al., 2009; Xue et al., 2015). Sb causes a deficiency in essential and trace metals in plants, and this decrease also results in a reduction in photosynthesis (Grundon, 2006). Sb-induced Fe, K, and Mg reduction is a major reason for the reduction in photosynthesis and subsequent growth of plants (Zhao et al., 2001; Ortega et al., 2017). Sb also causes damage to thylakoid and chloroplast, which causes cytotoxicity resulting in a serious reduction in photosynthetic efficiency (Paoli et al., 2013). Sb stress decreases chlorophyll synthesis owing to a reduction in Mg and Fe uptakes, and it also induces toxic effects on the photosynthetic apparatus, thereby causing a substantial reduction in photosynthesis.

## Effect of Sb toxicity on membrane permeability and lipid peroxidation

It has been recorded that ROS increases the malondialdehyde (MDA) contents that cause lipid peroxidation and instability of membranes (Shahid et al., 2017). Sb induces ROS production that interacts with membrane lipids and enhances lipid peroxidation leading to loss of membrane integrity (Rafiq et al., 2017). MDA and thiobarbituric acid reactive substance (TBARS) are major indicators of oxidative stress and peroxidation of lipids, and Sb stress substantially increases the MDA and TBARS contents. For instance, a linear increase in the MDA contents was noted in *Miscanthus sinensis* owing to Sb stress, which causes a significantly reduced membrane stability (Paoli et al., 2013; Xue et al., 2015). In another study, Chai et al. (2016) noted that MDA contents were significantly increased in plant roots and shoots under Sb stress. Conversely, Corrales et al. (2014) noted no significant increase in MDA accumulation in plants treated with SB; similarly, Feng et al. (2011a) also found no significant impact of Sb on MDA contents. Moreover, some authors noted a time-dependent variation in MDA levels; for instance, it has been recorded that the MDA contents in *Ficus tikoua* were significantly increased during early exposure, whereas at a later stage, they significantly reduced (Chai et al., 2017). Many other authors also noticed a substantial increase in lipid peroxidation and MDA production in various plants including sunflower, tomato, and maize (Ortega et al., 2017). Sb induces ROS production, which causes damage to cellular membranes and causes lipid peroxidation.

## Effect of Sb toxicity stress on oxidative stress and antioxidant activities

HMs induce oxidative damage that increases ROS, which causes serious damage to DNA, proteins, and lipids (Lu et al., 2018). A higher ROS production is a serious challenge under HM stress, and the production of ROS largely depends on the dose of HMs (Shahid et al., 2017; Ameen et al., 2019). Likewise, another HM, antimony toxicity also induces a serious increase in ROS production in a dose-dependent manner (Ortega et al., 2017). The high accumulation of Sb in plants also induces oxidative shock, which also results in overproduction of ROS (Peško et al., 2016). Sb stress also increases O_2_ (**Table 3**) production possibly by decreasing the antioxidant activities, particularly superoxide dismutase (SOD) (Pan et al., 2011). Moreover, Chai et al. (2016) noted that Sb in *Ficus tikoua* increased the SOD, peroxidase (POD), and (catalase) CAT activities, which resulted in no reduction in photosynthetic efficiency.

**Table 3:** Effects of antimony stress on growth, oxidative stress markers and antioxidant activities of different plant species.

Plant species	Antimony toxicity	Major effects	References
Rice	20 mg/L	Sb stress reduced the growth, biomass, salicylic acid and increased the ABA accumulation, MDA concentration and activities of APX, SOD and POD.	(Feng et al., 2020)
Ribbon fern	20 mg/kg	Sb toxicity reduced the chlorophyll contents, functions of ribosome, photosynthetic activity, and increased the MDA accumulation and ROS production.	(Xi et al., 2022)
Rice	10 mg/kg	Sb toxicity reduced the root and shoot growth, chlorophyll and carotenoid contents, electron transport, concentration of soluble sugars, and increases ROS production.	Zhu et al., (2022)
Maize	200 mg/kg	Sb stress reduced the leaf growth, stem length and stem diameter, root surface area, biomass, chlorophyll contents, and increased MDA accumulation, SOD, POD and CAT.	(Zhu et al., 2020)
Radish	100 mg/L	Sb stress reduced the seed germination, germination energy, germination rate and increased the accumulation of Sb in plant parts.	(Liang et al., 2018)
Sunflower	1 mM	Sb toxicity reduced the growth, biomass production, photosynthetic pigments, and increases the lipid per oxidation, total phenolics, GR, SOD, POD, AsA, and DHAR activity.	(Ortega et al., 2017)
Miscanthus	1000 μm	Sb stress reduced the leaf and root growth, chlorophyll contents, and increased MDA accumulation, POD activity and expression of stress responsive genes.	(Xue et al., 2015)
Water melon	8 mg/L	Sb toxicity reduced the chlorophyll contents and root and shoot growth and increased proline concentration as well as accumulation of Sb in plant parts.	(ARAGHI et al., 2014)
Maize	50 mg/L	Sb toxicity reduced the root length, root fresh and dry weight, and increased MDA and proline accumulation and APX, CAT, and GPOX activity as well as accumulation of Sb in plant parts.	(Vaculíková et al., 2014)

Sb stress also reduces the activity of non-enzymatic antioxidants; for instance, it has been reported that the activity of glutathione reductase (GR) was significantly decreased in response to Sb, resulting in a significant increase in ROS production (Feng et al., 2013a; Karacan et al., 2016). Plants activate antioxidant defense systems to cope with the effects of ROS, and Sb toxicity is substantially reduced by increasing antioxidant activities (Vaculíková et al., 2014). Xue et al. (2015) found that antioxidant activity in response to Sb toxicity varies according to plant species, concentration of Sb, and growing conditions. Similarly, Benhamdi et al. (2014) found that two plants, namely *Hedysarum pallidum* and *Lygeum spartum*, showed a significant difference in terms of antioxidant activities under Sb stress. *Lygeum spartum* plants showed significantly higher antioxidant activities, which indicate their higher ability to counter Sb stress, whereas maize plants exposed to Sb stress showed a marked reduction in POD and SOD activity indicating their lower ability to counter Sb stress (Pan et al., 2011). Moreover, alteration in antioxidant activities owing to Sb has been reported in many plants including rice, sunflower, and brassica (Feng et al., 2013b; Xi-Yuan et al., 2015; Ortega et al., 2017). The most abundant lower-molecular-weight thiols are glutathione synthetases (GSHs), and these thiols play an important role in the detoxification and sequestration of HMs against ROS (Foyer and Noctor, 2005). Likewise, Ortega et al. (2017) noted that exposure of sunflower plants to Sb stress significantly increased the GSH production and led to a substantial reduction in ROS production (Ortega et al., 2017). Ji et al. (2017) also noted that the Sb–thiol complex present in ryegrass roots ensures the immobilization and sequestration of Sb owing to GSH.

## Effect of Sb toxicity stress on osmolytes and hormones

Proline is an important osmolyte that plays a crucial role in plants under stress conditions. The role of proline in plants growing under Sb is poorly studied. Vaculíková et al. (2014) investigated the impact of Sb treatments on proline contents, and they found a significant increase in proline accumulation in plants exposed to Sb stress. They also found that increased Sb stress significantly increased the proline accumulation in roots and shoots of treated plants, which reduced the toxic effects of Sb stress (Jia et al., 2020). Moreover, Xue et al. (2015) also invested the variations in proline contents in *Miscanthus sinensis* under different levels of Sb stress. They found a linear increase in proline contents in miscanthus leaves with increasing Sb treatment. The increase in proline accumulation under Sb maintains a redox balance and alleviates Sb toxicity by maintaining the membrane integrity and ROS scavenging (Hayat et al., 2012). Ortega et al. (2017) also found that the redox status of sunflower was significantly increased under Sb owing to the increase in ascorbic acid (AsA) contents in the plants’ leaves. Conversely, Xi-Yuan et al. (2015) noted that Sb toxicity induces a reduction in AsA contents in *Brassica chinensis*. In short, plants accumulate stress protection through osmolytes and hormones to mitigate the adverse effects of Sb stress.

## Sb induced structural changes in plants

Sb stress also induces structural changes in the plant body, which is a major reason for Sb-induced reduction in plant growth. Stomata closure is an important physiological process, and Sb stress-induced closing of stomata reduces the carbon dioxide (CO_2_) intake, thus resulting in a significant reduction in photosynthesis (Romanowska et al., 2006). Daszkowska-Golec and Szarejko (2013) found that Sb causes the shrinkage of guard cells leading to the closure of stomata. Sb also damages the vascular bundle and xylem vessels and decreases the size of the meta-xylem, which reduces the upward movement of minerals and water from roots to shoots (Baruah et al., 2021). The ultrastructural changes caused by Sb also modify the water status of leaves, which decreases the water level in leaves and leads to the closure of stomata (Gowayed and Almaghrabi, 2013). Sb stress causes the disorganization of the chloroplast ultrastructure, which, in turn, reduces the photosynthesis (Baruah et al., 2021). Sb stress affects the structure of the chloroplast by degrading the structure of grana and stroma lamellae along with an increase in the quantity as well as dimension of plastoglobuli (Baruah et al., 2021). Cell wall plays an important role in the storage of HMs, and it works as the first barrier against the entry of HMs (Bora et al., 2020). The binding of Sb in the cell wall has been recognized as an important mechanism to detoxify (Feng et al., 2013b). To summarize, Sb toxicity also damages the vascular bundle, xylem vessels, cell wall, and chloroplast structure, which, in turn, induces substantial damage to plant performance.

## Soil–plant–human transfer of antimony

Sb has been recognized as a hazardous emerging pollutant, and it causes serious damage to humans and plants. It causes geno- and cytotoxicity and also carcinogenic diseases in humans (Bolan et al., 2022). Exposure of humans to Sb through oral, dermal, and inhalation causes serious effects on humans (Bagherifam et al., 2019). Sb disturbs the enzyme activity and adversely affects the heart, liver, kidney, and lungs (Wang et al., 2018). Sb inhalation can lead to lung cancer, and it also causes developmental, genotoxic, neurological, and reproductive abnormalities (US-PHS, 1992). A health risk noted that the hazard quotient (HQ) values of Sb in vegetables ranged from 1.61 to 3.33, and an HQ value higher than 1 can cause serious health risks (Zeng et al., 2015). The use of vegetables with HQ values greater than 1 caused serious health issues in the people of the Xikuangshan mine in Hunan, China (Zeng et al., 2015). Moreover, Sb contamination generally exists with As toxicity, which causes serious health issues in humans (Nishad and Bhaskarapillai, 2021).

Consumption of Sb-contaminated foods is a major reason for Sb entry into the human body (Pascaud et al., 2014). The excessive intake of Sb causes cancer and health, pulmonary, and renal failure in humans (WHO, 2003). In addition, Sb also causes lung, heart, and gastrointestinal diseases (Cooper and Harrison, 2009). Sb toxicity in humans largely depends on the dose, duration, the pathway of entry, sex, age, genetic traits, and occupational exposures (Cooper and Harrison, 2009). Exposure to atmospheric Sb (9 mg/m^3^) can cause eye, dermal, and lung irritation in humans (Cooper and Harrison, 2009). Edible plants growing in Sb-contaminated soils accumulate Sb in their tissues higher than the allowable level. Crops and vegetables have the capacity to accumulate Sb, which causes potential threats to humans (Pierart et al., 2015).

Zeng et al. (2015) performed an analysis of vegetable samples growing around the Sb-mining area in China. They found that the HQ indicated that the health risks for humans caused by Sb are much higher, and substantial measures are needed to control this problem. China is the largest Sb-mining country and is building Sb in its many regions. He and Yang (1999) found higher Sb levels in water and air near the largest mining area of Xikuangshan, China. Cen et al. (2007) also noted that long-term exposure of the residents of Guizhou, China to Sb stress caused liver cirrhosis, which caused many deaths in this region. Similarly, a higher level of Sb (16 mg/kg) was noted on the hairs of Xikuangshan residents (Wu et al., 2011) owing to eating Sb-contaminated foods. Milk is also a major reason for Sb entry into humans owing to the use of Sb-contaminated fodder by animals (Cooper and Harrison, 2009). Similarly, eating rice, vegetables, and wheat growing in Sb-contaminated areas is also a major reason for Sb entry into the human body (Ren et al., 2014; Cui et al., 2015). In conclusion, Sb accumulation must be minimized in vegetables and crops to reduce its entry into the human body.

## Sb remediation strategies

### Immobilization of Sb using soil amendments

**Figure 3:**
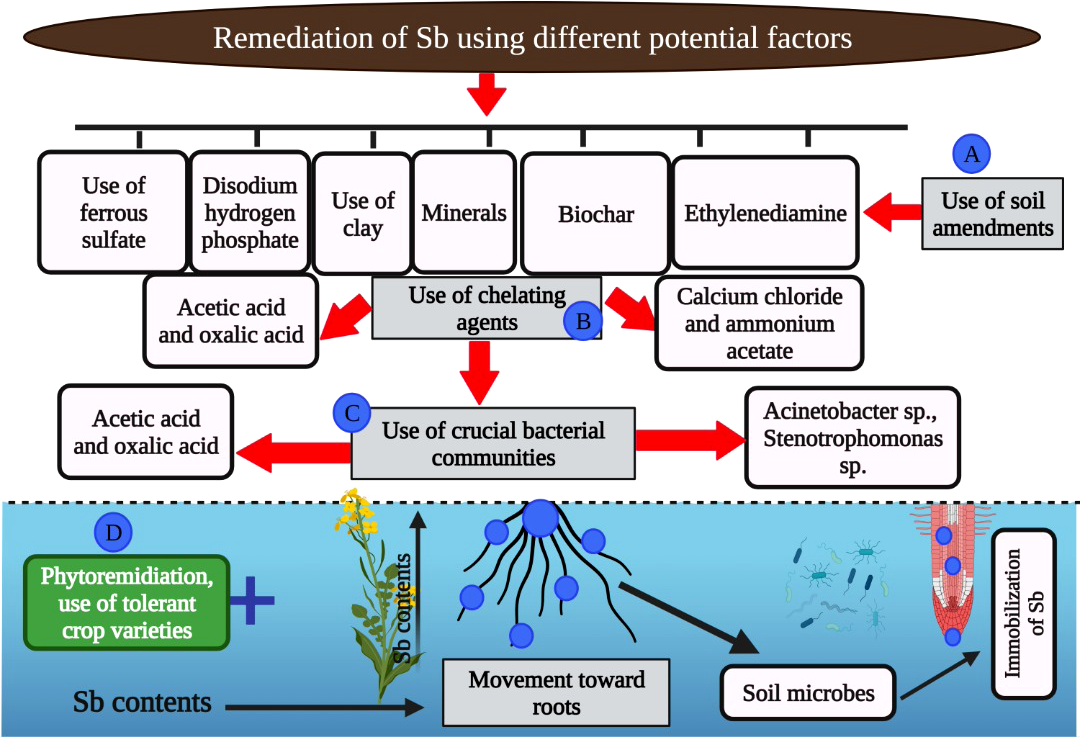
The role of various amendments to mitigate the Sb toxicity in plants. The various carbon based amendments (biochar), chemical treatments (ferrous sulphate, acetic acid, oxalic acid, ammonium acetate, and ethylenediamine) and microbes can cause immobilization of Sb which in turn reduce the toxic effects of Sb on plants.

The application of various materials, including biochar, clay mineral, fly ash, and organic compost (**Figure 3**), is considered an important practice to reduce the flow as well as availability of heavy metals including Sb (Palansooriya et al., 2020; Bolan et al., 2022). The chemical additives are considered to have better immobilization potential as compared to other additives (Silvani et al., 2019). Biochar application increases Sb immobilization; however, the application of biochar to Sb-contaminated soils needs further consideration (Rinklebe et al., 2020; Hua et al., 2021). Different authors also found appreciable Sb immobilization with the use of ferrous sulfate (Almås et al., 2019). Biochar also modifies the Fe–Mn immobilized Sb, therefore reducing the phyto-availability of Sb in Sb-contaminated soils (Wang et al., 2019). Moreover, Fe-modified biochar in agricultural soils also increased soil fertility and Sb retention in soil (Warnken et al., 2017). In another study, Zhang et al. (2021) noted that ammonium sulfate increased the bioavailability of Sb compared with the control owing to the fact that ammonium sulfate–induced higher pH increased the release of bound and organic Sb from soil. However, after 120 days of study, Sb bioavailability was reduced owing to increased Sb affinity for iron oxide present in the soil. The application of various amendments results in the higher retention of Sb in soils owing to the fact that change in soil pH after the application of these amendments can increase the bioavailability of Sb in soil (Wuana and Okieimen, 2011).

### Mobilization of Sb using soil leaching

Different chelating agents include acetic acid (AA), ammonium oxalate (AO), ammonium acetate (AA), calcium chloride, citrate, ethylenediaminetetraacetic acid (EDTA), ethylenediamine tetra (methylene phosphonic acid), and polyacrylic acid (Filella and Williams, 2012). It has been recorded that a lower amount of Sb was leached from soils using gentle extractants; however, higher leaching of Sb was achieved with organic extractants (Filella and Williams, 2012). Tan et al. (2018) subjected various Sb-contaminated soils (lightly, moderately, and highly contaminated) to different extractants. They noted that the efficacy of different extractants in leaching Sb from the soils decreased in the following order: citric acid, tartaric acid, EDTA, hydrochloric acid, disodium hydrogen phosphate, and calcium chloride. In another study, it was noted that water and disodium hydrogen phosphate substantially mobilized the Sb and reduced its toxic effects (Tan et al., 2018).

## Phytoremediation and microbial remediation of Sb-contaminated soils

Phytoremediation is considered an economical approach to remediate Sb-contaminated soils (Antoniadis et al., 2021; Prabha et al., 2021). It is a green and environmentally friendly approach compared with other methods to carry out the mobilization as well as immobilization of Sb (Gunarathne et al., 2020). Müller et al. (2013) performed a study for 7 weeks where they spiked the soil with various levels of Sb (5, 10, and 16 mg/kg) and studied its impact on the plant species *Pteris vittate*. They found that a significant amount of Sb was taken by plants grown under Sb stress. Similarly, sorghum plants grown under Sb also showed a significant increase in Sb accumulation in roots and the translocation of sorghum plants (Müller et al., 2013; Zand and Heir, 2020; Zand et al., 2020). Translocation factor >1 is considered a good indicator of phytoremediation (Antoniadis et al., 2021). Qi et al. (2011) compiled data from 31 different plant species, and they found that all species significantly uptake Sb, and many plants such *Barbarea verna*, *Sorghum bicolor* (sorghum), and *Nicotiana* showed TF>1.

Microbes are considered a crucial factor to remediating Sb-contaminated soils. Microbes present in soil reduce the toxicity and mobility of Sb by bio-reduction and bio-oxidation and changing Sb properties (Jeyasundar et al., 2021). Different Sb-oxidizing bacteria including *Acinetobacter, Stenotrophomonas, Comamonas, Shinella, Hydrogenophaga, Variovorax, Variovorax*, and *Flavihumibacter stibioxidans* have been identified to facilitate the oxidation of Sb (III) into Sb (II) (He et al., 2019a). Moreover, two strains of bacteria (*Shinella* and *Ensifer*) isolated from the Sb-contaminated soil showed significant oxidation of Sb in agar media with and without extract (Choi et al., 2017). Similarly, some bacterial strains including *Bacillales* also induced the transformation of Sb (V) into Sb (III) under anoxic conditions (Lai et al., 2018). A new strain of bacteria belonging to *Sinorhizobium* reduced the Sb (V) into Sb (III) under aerobic conditions (Nguyen and Lee, 2014). The production of monomethyl, dimethyl, and trimethyl Sb by different microbial communities has been well recognized (Hartmann et al., 2003; He et al., 2019b). Nonetheless, no information is needed regarding the role of amendments to immobilize the microbial methylated Sb in soils. In another study, Xi et al. (2022) reported that AMF maintains the function of ribosome and photosynthetic activities and counters the Sb toxicity by decreasing ROS production. In short, microbes cause oxidation of Sb, which, in turn, reduces the Sb retention in soil and its toxic effects on plants.

## Use of plant additives to reduce Sb toxicity

The external addition of selenium and silicon can also ameliorate Sb-contaminated soils by reducing the Sb uptake by crops. Selenium possesses an excellent potential to alleviate Sb toxicity by increasing antioxidant activities, regulating nutrient uptake, and inhibiting Sb uptake (Feng et al., 2011b; Feng et al., 2013a). Similarly, Huang et al. (2012) also found that Si also appreciably reduced the uptake as well as the toxicity of Sb in plants. Likewise, Qiang et al. (2017) also reported that Si application appreciably reduced the Sb toxicity in rice plants decreasing Sb uptake and increasing antioxidant activities. Moreover, Luo et al. (2021) also found that salicylic acid (SA) reduced the Sb-induced oxidative damage and MDA production by increasing proline, soluble sugar accumulation, and antioxidant activities and decreasing the Sb uptake. The foliar application of brassinosteroids significantly decreased the Sb-induced lipid peroxidation and Sb-induced oxidative damage by increasing enzymatic activities and proline accumulation (Wu et al., 2019). Additionally, in another study, it was found that nitrate (NO^3-^) application appreciably reduced the Sb availability by increasing the affinity of Sb for iron oxides. These authors also found that NO_3-_ also inhibited the reduction dissolution of iron minerals and resulted in a substantial reduction in Sb availability (Zhang et al., 2021).

An integrated approach uses different remediation strategies to remediate Sb-contaminated soils (Sun et al., 2020). Different authors around the globe used integrated approaches and obtained appreciable results to reduce the toxicity of Sb (Chirakkara et al., 2015; Sánchez et al., 2020; Girolkar et al., 2021). Couto et al. (2015) used phytoremediation along with electrokinetics and a phosphorus amendment for the removal of Sb from *Brassica* plants. They found that the addition of phosphorus reduced the Sb uptake by 30% in *Brassica* plants and 25% in ryegrass. Moreover, they also found that phosphorus amendment increases the Sb desorption from soil owing to the presence of the ionic repulsion between Sb and phosphate ions (Couto et al., 2015). In another study, Zand et al. (2020) studied the impact of titanium dioxide (TiO_2_) nanoparticles and sorghum bicolor on the remediation of Sb-contaminated soil. They found that TiO_2_ nanoparticles (0–1000 mg/kg) substantially improved biomass productivity and reduced Sb uptake and translocation (Zand and Heir, 2020). In another study, Zand et al. (2020) reported that TiO_2_–NP significantly improved the germination, chlorophyll contents, and plant biomass by reducing the Sb uptake and accumulation.

## Conclusion and future prospects

Antimony is a non-essential metal for plants and humans, and it is becoming a challenging heavy metal due to its anthropogenic activities. The entry of Sb into human food such as plants by growing them in Sb-contaminated soils is a major reason for Sb entry into the human body. Sb causes lung, heart, and gastrointestinal diseases. Sb stress also causes damaging effects on plants. Likewise, it reduces seed germination and root and shoot growth, causes damage to the photosynthetic apparatus, reduces membrane stability and nutrient uptake, and induces oxidative stress. Moreover, Sb toxicity also reduces membrane stability and disturbs osmolyte accumulation, water relations, and hormonal balance resulting in a substantial loss in plant growth. However, limited research is conducted on the toxic effects of Sb and possible solutions to increase crop production in Sb-contaminated soils. Sb toxicity negatively affects seed germination; however, it is not well explored how Sb toxicity reduces germination by affecting the various processes involved in seed germination. Sb toxicity substantially reduces nutrient uptake; nonetheless, limited information is available in the literature about this aspect. The toxic effects of Sb on nutrient uptake, nutrient channels, and transporters involved in nutrient uptake must be explored. The toxic effects of Sb on photosynthesis are well studied; however, it should also be explored in detail how Sb affects the photosynthesis and photosynthetic apparatus. Hormones and osmolytes play appreciably against abiotic stress tolerance. In the literature, limited studies are available about the toxic effects of Sb on the hormonal cross. Therefore, it is suggested that more detailed studies must be performed to determine the effect of Sb on various hormones and osmolyte accumulation to mitigate the adverse effects of Sb toxicity. The toxic effects of seed yield, seed quality, and plant reproductive characteristics must be explored, which will help to develop the appropriate measures to minimize the toxic effects of Sb.

The tolerance mechanism of Sb toxicity in plants is not well explored; therefore, there is a dire need to explore the tolerance mechanism of Sb toxicity in plants. It would be fascinating to determine how plants tolerate Sb for their survival. The various genes and stress-responsive proteins must be identified in plants to minimize the hazardous effects of Sb toxicity. The present increase in omics, metabolomics, proteome, and transcriptome techniques will allow exploring the Sb tolerance mechanisms in plants. The potential role of nanoparticles and various carbon-based amendments, hormones, and osmolytes should be explored to minimize the toxic effects of Sb in plants and humans. The microbial interaction is studied under controlled conditions; thus, more studies are needed to determine the microbial at field studies under Sb-contaminated soils. Lastly, the role of various amendments is studied under the pot experiment, and therefore, it is mandatory to conduct pilot plot studies to explore their roles to minimize Sb contamination.

## Author contributions

Conceptualization: HT and GH; writing—original draft: HT and GH; writing—review and editing: GM, JX, AM, GX, SU and YL. All authors contributed to the article and approved the submitted version.

## Funding

This work was supported and funded by the National Key R&D Program of China (2016YFD0300208); National Natural Science Foundation of China (41661070); Consulting Research Project of Chinese Academy of Engineering (2017-XY-28-03); Key Project of Hunan Provincial Department of Education (20A278); Agricultural Science and Technology Innovation Fund Project of Hunan Province: Research on Safe Utilization of Farmland with Excessive Heavy Metals in Tin Mines (2020cx84); and Hunan High-tech Industry Science and Technology Innovation Leading Plan Project: Research and Application of Key Technologies for Heavy Metal Reduction and Safe Production in Agricultural Water and Soil Environment (2020NK2001).

## Conflict of interest

Author JX was employed by Loudi Liancheng Hi-Tech Agricultural Development Co. LTD.

The remaining authors declare that the research was conducted in the absence of any commercial or financial relationships that could be construed as a potential conflict of interest.

## Publisher’s note

All claims expressed in this article are solely those of the authors and do not necessarily represent those of their affiliated organizations, or those of the publisher, the editors and the reviewers. Any product that may be evaluated in this article, or claim that may be made by its manufacturer, is not guaranteed or endorsed by the publisher.
